# Shared Functional Representation and Genomic Region Association Analysis of Longitudinal Phenotypes in Rice

**DOI:** 10.3390/e28101091

**Published:** 2026-10-07

**Authors:** Yuejiao Ji, Xiaoping Chen

**Affiliations:** 1 School of Mathematics and Statistics, Fujian Normal University, Fuzhou 350117, China; qbx20220076@yjs.fjnu.edu.cn; 2 School of Computing and Data Science, Fujian Provincial Key Laboratory of Big Data Mining and Applications, Fujian University of Technology, Fuzhou 350118, China

**Keywords:** functional data analysis, genomic region association analysis, information theory, longitudinal phenotypes, shared functional representation, spectral entropy

## Abstract

High-throughput phenotyping technologies enable plant growth to be measured repeatedly at high temporal resolution, providing rich information for studying the dynamic genetic basis of longitudinal traits. However, growth trajectories from repeated experiments contain both reproducible variation among lines and experiment-specific variation. In this study, we analyzed projected shoot area data from 356 rice lines measured in three independent experiments using an integrated genomic region association framework based on shared functional representation. We extracted the major longitudinal variation shared across experiments and characterized the covariance eigenspectra using spectral entropy and entropy-based effective rank. The first shared functional principal component explained 96.069% of shared variation, while the shared component had a normalized spectral entropy of 0.101 and an effective rank of 1.198. Regional association analysis using 200 kb genomic windows, with adjustment for population structure and leave-one-chromosome-out genetic relatedness, identified a region at 14.2–14.4 Mb on chromosome 10 that remained significant after false discovery rate correction (q=0.004). Further analyses showed time-varying regional effects and differences in association evidence across experiments, while the chromosome 10 signal was attenuated in an endpoint benchmark. Our analysis provides a method for linking longitudinal phenotypic variation shared across repeated experiments with localized genomic variation.

## 1. Introduction

Genetic improvement of crops requires accurate identification of the genetic basis of complex traits, while plant growth and development are continuous dynamic processes. Many important phenotypes, such as plant height, biomass, canopy structure, and response to external stress, are repeatedly or continuously expressed throughout the growth process. These traits are generally referred to as longitudinal traits [1]. In the past, the high cost of phenotyping and limited observation frequency restricted the collection of longitudinal phenotypes. Recently, advances in imaging, remote sensing, and automated sensing technologies have promoted the widespread use of high-throughput plant phenotyping platforms. These technologies allow researchers to repeatedly capture plant phenotypes in a non-destructive manner at high temporal resolution, providing more comprehensive growth trajectories [2,3]. High-frequency longitudinal phenotypes offer new opportunities to study the dynamic genetic basis of complex traits, but they also introduce new statistical challenges, including strong correlations between time points and potential systematic differences across experimental replicates. If the continuous temporal structure is ignored, dynamically changing traits may be reduced to static measurements, making it difficult to fully describe how phenotypic variation changes over time [4,5].

The genetic control of complex plant traits can also change over time. Existing quantitative trait loci (QTL) studies have shown that the same trait may be regulated by different genetic loci throughout distinct growth stages, and the magnitude of genetic effects may also vary during the growth process [6,7,8]. Endpoint phenotypes or separate association analyses at individual time points can identify some time-specific genetic signals, but they use only part of the information contained in a longitudinal trajectory. To make fuller use of the temporal process, several studies have analyzed phenotypes collected at multiple time points jointly and applied these approaches to crops such as sorghum, soybean, and sesame to investigate dynamic genetic signals [9,10,11]. How to effectively characterize the complete growth trajectory and further link these dynamic phenotypic changes with genetic variation remains an important problem in longitudinal plant phenotyping.

Functional Data Analysis provides a natural statistical framework for this purpose by treating the phenotype of the same individual over time as a function rather than a series of independent observations, thus preserving the continuous structure of the growth trajectory. Functional principal component analysis (FPCA) is a commonly used approach within this framework. It decomposes the functional covariance structure to identify the primary directions of variation in growth trajectories, and uses the corresponding eigenvalues to measure the variance explained by each direction [12]. When measuring longitudinal phenotypes in repeated experiments, the analysis must also distinguish variation shared across experiments from experiment-specific variation. Such decomposition is closely related to multilevel functional data analysis [13]. The proportion of variance explained (PVE) explained by the principal components is commonly used to assess whether the major variation is concentrated in a few functional directions. The complete eigenspectrum can additionally describe how the remaining variation is distributed across other orthogonal functional directions. After normalizing the eigenvalues by their sum, Shannon entropy summarizes the dispersion of the complete normalized spectrum [14], and entropy-based effective rank provides a corresponding measure of effective dimensionality [15]. Similar methods have been used to characterize phenotypic covariance structures in biological studies [16]. This suggests that for longitudinal phenotypes across multiple experiments, after extracting the shared functional components across experiments, an information-theoretic characterization of their functional covariance eigenspectrum can provide a supplement to the percentage of variance explained, and help describe the spectral concentration and low-dimensional structure of shared phenotypic variation.

While longitudinal phenotype representation describes dynamic phenotypic variation over time, association analysis also requires an appropriate representation of local genomic variation. Adjacent single-nucleotide polymorphisms (SNPs) in high-density genotype data are often correlated because of linkage disequilibrium. Therefore, jointly analyzing multiple correlated SNPs as a single genomic region can allow for the assessment of the relationship between local genetic variation and complex phenotypes at the regional scale [17]. Li et al. (2022) [18] combined functional data analysis with genomic regional information to study the association between longitudinal quantitative traits and genetic regions in rice, demonstrating the feasibility of conducting functional association analysis using both longitudinal phenotypic and regional genetic information. Their analysis was developed mainly in a single-experiment setting and used chromosome-level regions. However, for repeated experiments, it is also necessary to further distinguish reproducible growth variations across different experiments from experiment-specific variations, and to examine association analyses at a more localized genomic scale. Population structure and genetic relatedness within rice diversity panels also need to be appropriately accounted for in regional association analysis.

In this study, using the longitudinal projected shoot area (PSA) data from 356 rice lines measured in three independent experiments in Rice Diversity Panel 1, together with public genotype data [19,20], we aimed to develop an integrated genomic region association analysis framework based on shared functional representation. We extracted the major longitudinal phenotypic variation shared across experiments and characterized the eigenspectra of the shared and experiment-specific functional covariance components using Shannon entropy, normalized spectral entropy, and entropy-based effective rank. We then represented regional genetic variation using 200 kb local genomic windows and performed genome-wide regional association analyses after adjusting for population structure and genetic relatedness. For the candidate region identified from the genome-wide analysis, we further examined its dynamic regional effects and cross-experiment association patterns and evaluated its biological context using gene annotation and published rice QTL and genome-wide association study (GWAS) findings.

## 2. Materials and Methods

### 2.1. Plant Materials and Phenotypic Data

We analyzed the publicly available longitudinal projected shoot area (PSA) data for rice lines from the Rice Diversity Panel 1 (RDP1) [19,20]. The phenotypic data were obtained from the dataset reported by Campbell et al. [20]. Details of line selection, experimental design and implementation, data collection, and image processing in the source phenotyping study are described in that publication.

Briefly, in the source phenotyping study, three independent experiments, denoted E1, E2, and E3, were conducted from February to April 2016 at the Plant Accelerator facility of the University of Adelaide, Australia. Each experiment employed a partially replicated design, with 54 rice lines randomly selected to receive an additional plant replicate. During the plant growth period, an imaging system equipped with a visible-light red–green–blue (RGB) camera was used to image each potted rice plant daily from day 13 to day 33. Imaging angles included a top-down view and two side views spaced 90° apart. A total of 72,537 RGB images were obtained from the three experiments. After imaging, plant pixels were extracted using LemnaGrid software, and other non-plant pixels were removed. The PSA of rice was defined as the total number of plant pixels extracted from the three RGB images. In this study, we directly used publicly available derived PSA measurements without reprocessing the original RGB images. Previous studies have shown that PSA is strongly correlated with aboveground biomass and can be used to describe the dynamic growth process of aboveground parts of plants [20,21,22,23].

To ensure that the longitudinal phenotypes across the three experiments have consistent time domains, we aligned the longitudinal phenotypes from the three experiments to a common relative-day domain t=0,1,…,20. Let $P_{ier}\left(t_{j}\right)$, e=1,2,3, $r\in N_{ie}$, and j=0,…,20, denote the PSA of the *r*th plant from rice line *i* in experiment *e* at time $t_{j}$. We first checked the completeness and validity of the raw PSA data. Plants with non-positive PSA observations were excluded, and valid positive PSA values were transformed using $\log_{10}$. When multiple valid plant replicates were available for the same rice line within the same experiment, we averaged their log-transformed observations. The longitudinal phenotype used in subsequent analyses was

(1) $$Y_{ie}\left(t_{j}\right)=\frac{1}{n_{ie}}\sum_{r\in N_{ie}}\log_{10}\;\left\{P_{ier}\left(t_{j}\right)\right\},$$

where $N_{ie}$ is the set of valid plant replicates for rice line *i* in experiment *e*, and $n_{ie}=\left|N_{ie}\right|$ is the number of such replicates. This aggregation produced one longitudinal trajectory for each rice line within each experiment and avoided giving rice lines with multiple valid plant replicates greater weight in the cross-experiment covariance estimation. After phenotypic quality control and matching with the genotype samples, 356 rice lines were retained for subsequent analyses.

Since PSA was observed at discrete time points, we used B-spline basis functions to obtain continuous growth trajectories [24]. Let $B\left(t\right)={\left\{B_{1}\left(t\right),\ldots ,B_{K_{B}}\left(t\right)\right\}}^{⊤}$ be a B-spline basis defined on [0,20]. The smoothed curve for rice line *i* in experiment *e* was represented as

(2) $${\hat{Y}}_{ie}\left(t\right)=B{\left(t\right)}^{⊤}{\hat{c}}_{ie},$$

where ${\hat{c}}_{ie}$ is the B-spline coefficient vector. It was estimated by penalized least squares with a second-derivative roughness penalty, i.e.,

(3) $${\hat{c}}_{ie}=\underset{c}{arg min}\left[\sum_{j}{\left\{Y_{ie}\left(t_{j}\right)-B{\left(t_{j}\right)}^{⊤}c\right\}}^{2}+\lambda c^{⊤}Rc\right],$$

where $R=\int_{0}^{20}B^{\prime \prime }\left(t\right)B^{\prime \prime }{\left(t\right)}^{⊤}\;dt$ is the second-derivative roughness penalty matrix, and λ is the smoothing parameter. We used $K_{B}=10$ cubic B-spline basis functions and set $\lambda =10^{-4}$. The resulting continuous phenotype curves were used for the shared functional representation and genomic region association analyses described below.

### 2.2. Genotype Data and Genomic Region Construction

We used the publicly available Rice Diversity Panel 1 (RDP1) 44K SNP genotype data described by Zhao et al. [19]. The RDP1 genotype dataset contains 36,901 SNPs distributed across 12 chromosomes. Diploid genotypes are coded as 0, 1, and 2 according to the number of minor alleles. The physical positions provided in the public genotype dataset follow the coordinate system of MSU Rice Genome Annotation Project Release 6, and the genomic windows in this study were constructed using these positions. We first matched phenotype and genotype samples by rice-line identifiers and placed all data objects in the same sample order. Markers were excluded if their missing rate exceeded 0.10, their minor allele frequency (MAF) was below 0.05, or they were completely missing, invariant, or invalidly coded. After quality control, a total of 29,515 SNPs were retained. For missing markers, values were imputed using the average allelic dose of that marker in valid samples.

Let $x_{im}$ denote the allele dosage of rice line *i* at the *m*th SNP after quality control and missing-value imputation. Before performing principal component analysis (PCA), each SNP is standardized to

(4) $${\tilde{x}}_{im}=\frac{x_{im}-{\bar{x}}_{m}}{s_{m}},$$

where ${\bar{x}}_{m}$ and $s_{m}$ are the sample mean and sample standard deviation of the *m*th SNP dosage, respectively. When two or more SNPs had identical genotype dosage vectors across all rice lines, only one representative vector was retained for local PCA and construction of the genomic relationship matrix (GRM) to avoid duplicate weighting. This procedure yielded 28,581 unique genotype vectors. The correspondence to the original SNP identifiers and physical positions was retained.

Unlike Li et al. [18], who analyzed each chromosome as a single gene region, we constructed non-overlapping local windows with a width of 200 kb according to SNP physical positions within each chromosome. The *w*th window was defined as $W_{w}=\left[a_{w},a_{w}+200,000\right)$, where $a_{w}$ is the starting position of the window. Windows did not cross chromosome boundaries. Only windows containing at least five SNPs that passed quality control were included in subsequent analyses. This resulted in 1673 testable primary windows. Furthermore, SNPs within the same region often exhibit strong correlation. To reduce local collinearity while retaining the main regional genetic variation, we applied PCA to the standardized genotype matrix within each window [25]. We retained the minimum number of local principal components (PCs) needed to explain at least 80% of the cumulative genotype variance, subject to a prespecified maximum of five components. If the first five principal components did not reach 80% cumulative PVE, all five components were retained and their actual cumulative PVE was recorded. Let the number of principal components retained in window *w* be $q_{w}$. The local genetic representation of rice line *i* was then

(5) $$g_{iw}={\left(g_{iw1},\ldots ,g_{iwq_{w}}\right)}^{⊤}.$$

### 2.3. Shared Functional Representation and Information-Theoretic Characterization of Longitudinal Phenotypes Across Experiments

The rice growth curves from the three independent experiments contain both longitudinal variation shared among rice lines and experiment-specific variation. To extract a low-dimensional representation reflecting the common growth changes in the lines from the longitudinal phenotypes of repeated experiments, we decomposed the smoothed longitudinal curves using a multilevel functional structure [13]. For notational simplicity, we write the smoothed curve ${\hat{Y}}_{ie}\left(t\right)$ from Section 2.1 as $Y_{ie}\left(t\right)$ in this subsection. We assumed

(6) $$Y_{ie}\left(t\right)=\mu \left(t\right)+\alpha_{e}\left(t\right)+Z_{i}\left(t\right)+D_{ie}\left(t\right),\;i=1,\ldots ,n,\;e=1,2,3,$$

where μ(t) is the overall mean growth function, $\alpha_{e}\left(t\right)$ is the fixed-effect function of the *e*th experiment, $Z_{i}\left(t\right)$ is the random functional component shared by the same rice line across experiments, and $D_{ie}\left(t\right)$ is the experiment-specific composite deviation. As the available plant replicates were averaged within each rice line and experiment, $D_{ie}\left(t\right)$ represents the remaining experiment-specific deviation for rice line *i* in experiment *e*. It may include variation specific to that rice line in that experiment, together with random variation that remains after averaging the plant replicates. In this study, $D_{ie}\left(t\right)$ is treated as a composite experiment-specific deviation rather than a separate plant-level random effect. To distinguish between shared variations and experiment-specific variations, we made the following assumptions.

- **Assumption 1:** $\sum_{e=1}^{3}\alpha_{e}\left(t\right)=0,\;E\left\{Z_{i}\left(t\right)\right\}=0,\;E\left\{D_{ie}\left(t\right)\right\}=0.$
- **Assumption 2:** For any s,t∈[0,20], $\mathrm{Cov}\{Z_{i}\left(s\right),D_{ie}\left(t\right)\}=0$, and, for $e\neq e^{\prime }$, $\mathrm{Cov}\{D_{ie}\left(s\right),D_{ie^{\prime }}\left(t\right)\}=0$.

Assumption 2 means that the shared random function is uncorrelated with the experimental specificity deviations, and that specificity deviations from different experiments are also uncorrelated. Under these assumptions, the cross-covariance between different experimental curves of the same rice line is generated only by the shared random function $Z_{i}\left(t\right)$.

Expanding each function in Equation (6) onto the same B-spline basis as in Section 2.1, we have

(7) $$c_{ie}=b_{\mu }+b_{\alpha ,e}+z_{i}+d_{ie},$$

where $c_{ie}$, $b_{\mu }$, $b_{\alpha ,e}$, $z_{i}$, and $d_{ie}$ are the B-spline coefficient vectors of $Y_{ie}\left(t\right)$, μ(t), $\alpha_{e}\left(t\right)$, $Z_{i}\left(t\right)$, and $D_{ie}\left(t\right)$, respectively. Under the constraint $\sum_{e=1}^{3}b_{\alpha ,e}=0$, we can use the integral of the squared errors for all lines and experimental curves to estimate the overall mean and the experimental fixed effects, and obtain the residual coefficients

$${\hat{r}}_{ie}=c_{ie}-{\hat{b}}_{\mu }-{\hat{b}}_{\alpha ,e}.$$

For two different experiments $e\neq e^{\prime }$, let the sample cross covariance of the residual coefficients be

(8) $${\hat{C}}_{ee^{\prime }}=\frac{1}{n-1}\sum_{i=1}^{n}\left({\hat{r}}_{ie}-{\bar{r}}_{e}\right){\left({\hat{r}}_{ie^{\prime }}-{\bar{r}}_{e^{\prime }}\right)}^{⊤},$$

where ${\bar{r}}_{e}=\frac{1}{n}\sum_{i=1}^{n}{\hat{r}}_{ie}$. The covariance of the shared random function in the B-spline coefficient space is estimated by the symmetric mean of the three pairs of experimental cross covariances, that is

(9) $${\hat{\Sigma }}_{Z}=\frac{1}{6}\left({\hat{C}}_{12}+{\hat{C}}_{12}^{⊤}+{\hat{C}}_{13}+{\hat{C}}_{13}^{⊤}+{\hat{C}}_{23}+{\hat{C}}_{23}^{⊤}\right).$$

In addition, for experiment *e*, we can estimate the residual coefficient covariance matrix by

(10) $${\hat{S}}_{e}=\frac{1}{n-1}\sum_{i=1}^{n}\left({\hat{r}}_{ie}-{\bar{r}}_{e}\right){\left({\hat{r}}_{ie}-{\bar{r}}_{e}\right)}^{⊤}.$$

Combining with $\mathrm{Var}\left(z_{i}+d_{ie}\right)=\Sigma_{Z}+\Sigma_{D,e}$, we get the covariance estimate for the experiment-specific composite deviation as

(11) $${\hat{\Sigma }}_{D,e}={\hat{S}}_{e}-{\hat{\Sigma }}_{Z},\;e=1,2,3.$$

Let $J=\int_{0}^{20}B\left(t\right)B{\left(t\right)}^{⊤}dt$, denote the Gram matrix of the B-spline basis. Since finite sample estimation may produce negative eigenvalues, we perform a positive semidefinite correction on ${\hat{\Sigma }}_{Z}$ and ${\hat{\Sigma }}_{D,e}$ in the B-spline Gram inner product space. Specifically, for $A\in \{Z,D_{1},D_{2},D_{3}\}$, we eigendecomposed $J^{1/2}{\hat{\Sigma }}_{A}J^{1/2}$, truncated negative eigenvalues to zero, and transformed the corrected matrix back to the B-spline coefficient space. For notational simplicity, the corrected covariance matrices are still denoted by ${\hat{\Sigma }}_{Z}$ and ${\hat{\Sigma }}_{D,e}$ below.

Therefore, the covariance function of the shared random function can be estimated as

(12) $${\hat{C}}_{Z}\left(s,t\right)=B{\left(s\right)}^{⊤}{\hat{\Sigma }}_{Z}B\left(t\right).$$

We performed FPCA on ${\hat{C}}_{Z}\left(s,t\right)$. The estimated eigenvalues ${\hat{\lambda }}_{\ell }$ and eigenfunctions ${\hat{\psi }}_{\ell }\left(t\right)$ satisfy

$$\int_{0}^{20}{\hat{C}}_{Z}\left(s,t\right){\hat{\psi }}_{\ell }\left(s\right)\;ds={\hat{\lambda }}_{\ell }{\hat{\psi }}_{\ell }\left(t\right),\;{\hat{\lambda }}_{1}\geq {\hat{\lambda }}_{2}\geq \cdots \geq 0,$$

with the orthonormality condition

$$\int_{0}^{20}{\hat{\psi }}_{\ell }\left(t\right){\hat{\psi }}_{\ell^{\prime }}\left(t\right)\;dt=\left\{\begin{array}{cc} 1, & \ell =\ell^{\prime },\\ 0, & \ell \neq \ell^{\prime }. \end{array}\right.$$

We further characterized the eigenspectra of the shared and experiment-specific functional covariance components from an information-theoretic perspective. For $A\in \{Z,D_{1},D_{2},D_{3}\}$, let ${\hat{\lambda }}_{A1},\ldots ,{\hat{\lambda }}_{AR_{A}}$ denote all positive eigenvalues of the corresponding positive semidefinite covariance operator. These functional eigenvalues are equivalently the positive eigenvalues of $J^{1/2}{\hat{\Sigma }}_{A}J^{1/2}$. For the shared component, it follows that ${\hat{\lambda }}_{Z\ell }={\hat{\lambda }}_{\ell }$. In the numerical calculation, eigenvalues larger than $10^{-10}{\hat{\lambda }}_{A1}$ were treated as positive, and $R_{A}$ denotes the resulting positive eigenvalue rank. We normalized the positive eigenvalues as

(13) $$p_{A\ell }=\frac{{\hat{\lambda }}_{A\ell }}{\sum_{m=1}^{R_{A}}{\hat{\lambda }}_{Am}},\;\ell =1,\ldots ,R_{A}.$$

The Shannon entropy of the normalized eigenspectrum was then defined as [14]

(14) $$H_{A}=-\sum_{\ell =1}^{R_{A}}p_{A\ell }\log p_{A\ell }.$$

We used the natural logarithm, so $H_{A}$ is measured in nats. As the covariance components may have different positive eigenvalue ranks, we also used the normalized spectral entropy

(15) $$H_{A}^{\mathrm{norm}}=\frac{H_{A}}{\log R_{A}},\;R_{A}>1.$$

For $R_{A}=1$, we set $H_{A}^{\mathrm{norm}}=0$. According to [15], the effective rank based on entropy was $r_{\mathrm{eff},A}=\exp\left(H_{A}\right)$. Here, $p_{A1}$ measures the proportion of positive spectral mass in the first functional direction, whereas $H_{A}$ and $r_{\mathrm{eff},A}$ summarize the distribution of variation over the complete normalized positive eigenspectrum. Thus, the first component PVE and the information theoretic summaries describe different aspects of the same eigenspectrum. The latter remain deterministic functions of the complete normalized PVE spectrum.

To investigate how the remaining positive spectral mass is distributed after separating the first component, we further decomposed each complete positive eigenspectrum into its first component and remaining components. This supplementary decomposition is independent of the number of shared functional principal components retained later for genomic region association. For $R_{A}>1$, we defined the conditional tail weights as $q_{A\ell }=p_{A\ell }/\left(1-p_{A1}\right),\;\ell =2,\ldots ,R_{A}.$ The conditional tail entropy and the corresponding tail effective rank were defined as

$$H_{A,\mathrm{tail}}=-\sum_{\ell =2}^{R_{A}}q_{A\ell }\log q_{A\ell },\;r_{\mathrm{eff},A}^{\mathrm{tail}}=\exp\;\left(H_{A,\mathrm{tail}}\right).$$

With h(p)=−plogp−(1−p)log(1−p), the total spectral entropy can be written as

(16) $$H_{A}=h\left(p_{A1}\right)+\left(1-p_{A1}\right)H_{A,\mathrm{tail}}.$$

Through this decomposition, we can distinguish the distribution of the spectral mass assigned to the first component from the distribution of the remaining mass in other positive functional directions. For supplementary time domain diagnostics, let ${\hat{\psi }}_{A\ell }\left(t\right)$ denote the eigenfunction corresponding to ${\hat{\lambda }}_{A\ell }$. We defined the time resolved variance contributed by the remaining positive components as $v_{A,\mathrm{tail}}\left(t\right)=\sum_{\ell =2}^{R_{A}}{\hat{\lambda }}_{A\ell }{\hat{\psi }}_{A\ell }^{\;2}\left(t\right),$ and normalized it over the full relative-day domain as

$$d_{A,\mathrm{tail}}\left(t\right)=\frac{v_{A,\mathrm{tail}}\left(t\right)}{\int_{0}^{20}v_{A,\mathrm{tail}}\left(u\right)\;du}.$$

Relative day 0–20 was partitioned into three prespecified intervals of equal length. The tail variance share of each interval was obtained by integrating $d_{A,\mathrm{tail}}\left(t\right)$ over that interval.

Lower spectral entropy indicates that functional variation is more concentrated in fewer orthogonal directions, while $r_{\mathrm{eff},A}$ provides an entropy-based summary of the effective dimensionality of the covariance eigenspectrum. All information theoretic summaries and supplementary tail diagnostics were calculated from the complete positive eigenspectrum after the positive semidefinite correction and before any component-retention rule was applied. For the subsequent genomic region association analysis, we retained the minimum number of shared functional principal components needed to explain at least 95% of the cumulative variance, with a prespecified maximum of five components.

Furthermore, to estimate the shared random function for each rice line, we let ${\hat{r}}_{i}={\left({\hat{r}}_{i1}^{⊤},{\hat{r}}_{i2}^{⊤},{\hat{r}}_{i3}^{⊤}\right)}^{⊤}$. Under Assumption 2, we estimated the shared coefficients using linear optimal prediction:

(17) $${\hat{z}}_{i}=\left(\begin{array}{ccc} {\hat{\Sigma }}_{Z} & {\hat{\Sigma }}_{Z} & {\hat{\Sigma }}_{Z} \end{array}\right){\hat{\Sigma }}_{r}^{+}{\hat{r}}_{i}.$$

where the three diagonal blocks of ${\hat{\Sigma }}_{r}$ are ${\hat{\Sigma }}_{Z}+{\hat{\Sigma }}_{D,e}$, and the off-diagonal blocks are all ${\hat{\Sigma }}_{Z}$, with the superscript + denoting the Moore–Penrose generalized inverse. It leads to

(18) $${\hat{Z}}_{i}\left(t\right)=B{\left(t\right)}^{⊤}{\hat{z}}_{i}.$$

If ${\hat{\psi }}_{\ell }\left(t\right)=B{\left(t\right)}^{⊤}{\hat{a}}_{\ell }$, then, the corresponding functional principal component score is

(19) $${\hat{\eta }}_{i\ell }=\int_{0}^{20}{\hat{Z}}_{i}\left(t\right){\hat{\psi }}_{\ell }\left(t\right)\;dt={\hat{a}}_{\ell }^{⊤}J{\hat{z}}_{i}.$$

### 2.4. Genomic Region Association Analysis

To adjust for population structure and genetic relatedness among rice lines, we separately employed genome-wide principal components and the leave-one-chromosome-out (LOCO) genomic relationship matrix. For any two SNPs, let *r* denote the Pearson correlation between their standardized genotype dosages. We used a threshold of $r^{2}=0.20$ for linkage disequilibrium (LD) pruning, retaining a total of 1090 relatively independent SNPs. PCA was then applied to these SNPs, and the top five principal components were included as fixed covariates in the region association model to control population structure [26]. The LD-pruned marker set was used for global PCA in the primary analysis. In contrast, the primary LOCO GRM was constructed from all unique genotype vectors that passed quality control after excluding the chromosome containing the tested window.

Global principal components primarily describe population structure on a broad scale, whereas more detailed genetic relatedness among lines can be modeled through GRM. For a window located on chromosome *c*, all SNPs on chromosome *c* were excluded to reduce proximal contamination [27,28]. Let $X^{\left(-c\right)}$ be the $n\times M_{-c}$ matrix of unique genotype vectors after excluding chromosome *c*, with each SNP standardized across rice lines. The LOCO GRM for chromosome *c* was defined as

(20) $$K^{\left(-c\right)}=\frac{1}{M_{-c}}X^{\left(-c\right)}X^{(-c)⊤},$$

where $M_{-c}$ is the number of markers used to construct the matrix. For window *w* and the *ℓ*th shared functional principal component, we fitted the linear mixed model

(21) $${\hat{\eta }}_{i\ell }=\beta_{0,c\ell }+p_{i}^{⊤}\gamma_{c\ell }+g_{iw}^{⊤}b_{w\ell }+u_{i,c\ell }+\varepsilon_{i,c\ell },$$

where $\beta_{0,c\ell }$ is the intercept, $\gamma_{c\ell }$ is the vector of fixed-effect coefficients, $p_{i}$ contains the first five global-PC scores for rice line *i*, $g_{iw}$ contains the local-PC scores of rice line *i* in window *w*, and $b_{w\ell }$ is the corresponding vector of regional effect coefficients. We assume that the polygenic random-effect vector $u_{c\ell }={\left(u_{1,c\ell },\ldots ,u_{n,c\ell }\right)}^{⊤}$ and the residual vector $\varepsilon_{c\ell }={\left(\varepsilon_{1,c\ell },\ldots ,\varepsilon_{n,c\ell }\right)}^{⊤}$ satisfy $u_{c\ell }∼N\;\left(0,\tau_{c\ell }K^{\left(-c\right)}\right)$, and $\varepsilon_{c\ell }∼N\;\left(0,\sigma_{c\ell }^{2}I_{n}\right)$, where $u_{c\ell }$ and $\varepsilon_{c\ell }$ are independent.

To avoid repeatedly estimating the variance components for each window on the same chromosome, we first fitted a null linear mixed model without the local window term for each shared functional principal component and each chromosome:

(22) $${\hat{\eta }}_{\ell }=X_{0}\theta_{c\ell }+u_{c\ell }+\varepsilon_{c\ell },$$

where ${\hat{\eta }}_{\ell }={\left({\hat{\eta }}_{1\ell },\ldots ,{\hat{\eta }}_{n\ell }\right)}^{⊤}$, $X_{0}=\left[1_{n},\;P\right]$ is the fixed-effect design matrix, $1_{n}$ is a vector of ones, P is the matrix containing the first five global-PC scores, and $\theta_{c\ell }$ is the corresponding fixed-effect coefficient vector. The covariance matrix under the null model is

(23) $$V_{c\ell }=\tau_{c\ell }K^{\left(-c\right)}+\sigma_{c\ell }^{2}I_{n}.$$

The variance components $\tau_{c\ell }$ and $\sigma_{c\ell }^{2}$ were estimated by restricted maximum likelihood (REML) [29]. All main windows on the same chromosome are fixed using the ${\hat{\tau }}_{c\ell }$, ${\hat{\sigma }}_{c\ell }^{2}$ and ${\hat{V}}_{c\ell }$ obtained from the null model, and the variance components are not repeatedly estimated for each local window.

Under the estimated null covariance, we defined the weighted projection matrix after removing the fixed effects of $X_{0}$ as

(24) $${\hat{R}}_{c\ell }={\hat{V}}_{c\ell }^{-1}-{\hat{V}}_{c\ell }^{-1}X_{0}{\left(X_{0}^{⊤}{\hat{V}}_{c\ell }^{-1}X_{0}\right)}^{-1}X_{0}^{⊤}{\hat{V}}_{c\ell }^{-1}.$$

Let $G_{w}={\left(g_{1w},\ldots ,g_{nw}\right)}^{⊤}$. For the window *w*, the corresponding region score vector and information matrix were

(25) $$U_{w\ell }=G_{w}^{⊤}{\hat{R}}_{c\ell }{\hat{\eta }}_{\ell },\;I_{w\ell }=G_{w}^{⊤}{\hat{R}}_{c\ell }G_{w}.$$

Then, under the condition of the fixed null-model variance component estimates, we can get the generalized least-squares estimate of the regional effect as

(26) $${\hat{b}}_{w\ell }=I_{w\ell }^{+}U_{w\ell },$$

with estimated covariance $\hat{\mathrm{Var}}\left({\hat{b}}_{w\ell }\right)=I_{w\ell }^{+}$.

For each window, what we are interested in is whether the local genetic variation represented by that window remained associated with the *ℓ*th shared functional principal component score after adjustment for global population structure and LOCO genetic relatedness. Since the window *w* is jointly represented by $q_{w}$ local principal components, this problem can be reformulated as testing whether the regional effect coefficients corresponding to these local principal components are all zero, that is,

(27) $$H_{0,w\ell }:b_{w\ell }=0\;\mathrm{vs}.\;H_{1,w\ell }:b_{w\ell }\neq 0.$$

To test this, we used the regional Wald statistic

(28) $$Q_{w\ell }=U_{w\ell }^{⊤}I_{w\ell }^{+}U_{w\ell }.$$

Let $r_{w\ell }=\mathrm{rank}\left(I_{w\ell }\right)$ be the rank of the local information matrix. Under the null hypothesis, we have $Q_{w\ell }\overset{a}{∼}\chi_{r_{w\ell }}^{2}$. For the primary genome-wide scan, the Benjamini–Hochberg (BH) procedure was applied across all 1673 primary-window *p*-values to control the false discovery rate (FDR) [30]. An adjusted q<0.05 was used as the criterion for selecting candidate regions.

### 2.5. Dynamic Regional Effects and Cross-Experiment Assessments

The region test can identify local genomic regions associated with the shared longitudinal phenotype representation, but it cannot directly reflect how regional effects change over time. For candidate regions identified through genome-wide FDR screening, we further combined the local genetic directions represented by local PCA with the shared functional principal component directions of the phenotype to reconstruct their dynamic regional effects.

Let $s_{wm}$ denote the physical position of SNP *m* in window *w*, and let $v_{wmk}$ denote its loading on the *k*th local principal component. For the *ℓ*th shared functional principal component, the projected regional coefficient at this location was

(29) $${\hat{\theta }}_{wm\ell }=\sum_{k=1}^{q_{w}}v_{wmk}{\hat{b}}_{wk\ell },$$

where ${\hat{b}}_{wk\ell }$ is the estimated effect of the *k*th local principal component in window *w* on the *ℓ*th shared functional principal component score. Combining these coefficients with the shared eigenfunctions ${\hat{\psi }}_{\ell }\left(t\right)$ gives the dynamic regional effect of window *w* at genomic position $s_{wm}$ and time *t*:

(30) $$\begin{array}{c} {\hat{\beta }}_{w}\left(s_{wm},t\right)=\sum_{\ell =1}^{L}{\hat{\theta }}_{wm\ell }{\hat{\psi }}_{\ell }\left(t\right), \end{array}$$

where *L* is the number of shared functional principal components retained in Section 2.3.

These quantities are the result of projecting window-level local principal component analysis effects onto representative physical location anchors; they are not marginal effects estimated independently for individual SNPs. To further assess the performance of these associations across different experiments, we conducted leave-one-experiment-out (LOEO) cross-experiment robustness assessments. In each LOEO fold, one experiment was treated as the held-out experiment and the other two were used as the training experiments. We re-estimated the shared function representations from Section 2.3 using the two training experiments and performed the main window region scan again following the method described in Section 2.4. For the held-out experiment, only its longitudinal phenotype data were used to estimate the experiment level mean offset. The adjusted curves were then projected onto the shared functional principal component directions estimated from the two training experiments, and a targeted regional test was performed for the same candidate window selected in the full-data analysis. Held-out assessments are not interpreted as independent replication.

The LOEO assessment can reflect the degree of association support for candidate regions in experiments not involved in shared representation estimation, but it cannot directly compare the region effects in three experiments. To this end, after identifying candidate regions based on the full dataset, we projected the longitudinal curves from each experiment onto the shared temporal direction. For the *ℓ*th shared functional principal component, define the projection score of rice line *i* in experiment *e* as $S_{ie\ell }=\langle Y_{ie}\left(t\right)-\hat{\mu }\left(t\right)-{\hat{\alpha }}_{e}\left(t\right),{\hat{\psi }}_{\ell }\left(t\right)\rangle$, where $\langle f,g\rangle =\int_{0}^{20}f\left(t\right)g\left(t\right)\;dt$ denotes the $L^{2}\left[0,20\right]$ inner product. Here, $\hat{\mu }\left(t\right)$, ${\hat{\alpha }}_{e}\left(t\right)$, and ${\hat{\psi }}_{\ell }\left(t\right)$ were estimated from the shared functional model fitted to all three experiments. For candidate window *w*, we fitted the joint cross-experiment linear mixed model

(31) $$S_{ie\ell }=\zeta_{e\ell }+p_{i}^{⊤}\gamma_{e\ell }+g_{iw}^{⊤}b_{e\ell }+a_{i\ell }+\varepsilon_{ie\ell },$$

where $\zeta_{e\ell }$ is the intercept for experiment *e*, $a_{\ell }={\left(a_{1\ell },\ldots ,a_{n\ell }\right)}^{⊤}∼N\;\left(0,\tau_{\ell }^{\left(H\right)}K^{\left(-c\right)}\right)$, and the residual vectors from the three experiments within the same line satisfy $\varepsilon_{i\ell }={\left(\varepsilon_{i1\ell },\varepsilon_{i2\ell },\varepsilon_{i3\ell }\right)}^{⊤}∼N\;\left(0,\Sigma_{\varepsilon ,\ell }\right)$, where $\Sigma_{\varepsilon ,\ell }$ is an unstructured 3×3 covariance matrix. Variance parameters were estimated by REML, and the fixed effects were estimated by generalized least squares under the estimated covariance structure.

To compare regional effects among the three experiments, we stacked the experiment-specific effect vectors as $b_{\ell }={\left(b_{1\ell }^{⊤},b_{2\ell }^{⊤},b_{3\ell }^{⊤}\right)}^{⊤}.$ For a general linear hypothesis

(32) $$H_{0}:Lb_{\ell }=0,$$

we used the Wald statistic

(33) $$W_{L}={\left(L{\hat{b}}_{\ell }\right)}^{⊤}{\left\{L\hat{\mathrm{Var}}\left({\hat{b}}_{\ell }\right)L^{⊤}\right\}}^{+}\left(L{\hat{b}}_{\ell }\right),$$

to test and used the rank of the contrast covariance matrix as the degrees of freedom for the $\chi^{2}$ approximation.

We primarily aimed to test whether the average regional effect across the three experiments was zero, whether there was heterogeneity in the regional effect across the experiments, and compared the regional effect in E3 with the average effect in E1 and E2. The corresponding null hypotheses were

$$\begin{array}{cc} H_{0}^{\mathrm{avg}}: & \;\frac{b_{1\ell }+b_{2\ell }+b_{3\ell }}{3}=0,\\ H_{0}^{\mathrm{het}}: & \;b_{1\ell }=b_{2\ell }=b_{3\ell },\\ H_{0}^{E3}: & \;b_{3\ell }-\frac{b_{1\ell }+b_{2\ell }}{2}=0. \end{array}$$

We also tested the regional effect against zero separately within each experiment. The three experiment-specific *p*-values were adjusted using the Holm procedure [31].

### 2.6. Finite-Sample Diagnostics

The regional tests in Section 2.4 use an asymptotic $\chi^{2}$ distribution to obtain window-level *p*-values. To examine the calibration of this approximation across the primary scan, we evaluated the quantile–quantile (QQ) plots for all 1673 primary window *p*-values and separately by their observed effective degrees of freedom. In addition to genome-wide calibration diagnostics, we also performed candidate specific finite-sample diagnostics on candidate regions identified through primary genome-wide FDR screening.

Given the limited sample size and the genetic correlation among lines, we further conducted conditional parametric bootstrap analysis on the candidate regions identified through genome-wide FDR screening [32]. For the chromosome containing the candidate region and the corresponding shared functional principal component, we used the null model fitted in Section 2.4 and generated

$$y_{\ell }^{*(b)}∼N\;\left(X_{0}{\hat{\theta }}_{c\ell },{\hat{V}}_{c\ell }\right)$$

under the condition of fixed

$${\hat{V}}_{c\ell }={\hat{\tau }}_{c\ell }K^{\left(-c\right)}+{\hat{\sigma }}_{c\ell }^{2}I_{n}.$$

Across all bootstrap replicates, the candidate window, its local-PC representation, the global principal components, the LOCO GRM, and the null-model variance component estimates were kept fixed. The same regional statistic used in Section 2.4 was recalculated for each replicate to obtain $Q_{w\ell }^{*(b)}$. We performed B=20,000 bootstrap replicates. The empirical *p*-value was

(34) $${\hat{p}}_{\mathrm{emp}}=\frac{1+\sum_{b=1}^{B}1\;\left\{Q_{w\ell }^{*(b)}\geq Q_{w\ell }^{\mathrm{obs}}\right\}}{B+1},$$

where $Q_{w\ell }^{\mathrm{obs}}$ is the regional statistic from the observed data and 1{·} is the indicator function. Based on the number of bootstrap statistics exceeding $Q_{w\ell }^{\mathrm{obs}}$, we also calculated an exact Clopper–Pearson confidence interval for the corresponding binomial probability [33].

In addition, to examine whether the candidate regions were excessively influenced by any single line, we further conducted a leave-one-line-out analysis. We refitted the corresponding LOCO null models to the remaining lines and performed regional tests, while keeping the candidate regions, local-PC definitions, and global principal component column definitions unchanged. Through comparing the *p*-values of candidate windows and the direction of estimated regional effects across omissions, we can evaluate the stability to perturbations of individual rice lines.

### 2.7. Semisimulation Study

We conducted a semisimulation study based on the observed sample structure, genotype data, and functional covariance estimates to evaluate the empirical behavior of the analysis framework and its associated regional tests under prespecified regional effect structures. The simulated phenotype was generated from

(35) $$Y_{ie}^{*}\left(t\right)=\hat{\mu }\left(t\right)+{\hat{\alpha }}_{e}\left(t\right)+Z_{i}^{*}\left(t\right)+D_{ie}^{*}\left(t\right),$$

where

$$Z_{i}^{*}\left(t\right)=\left(\xi_{i}+\delta_{Z}Q_{i}\right){\hat{\psi }}_{1}\left(t\right),\;D_{ie}^{*}\left(t\right)={\tilde{D}}_{ie}\left(t\right)+\delta_{D,e}Q_{i}{\hat{\psi }}_{1}\left(t\right).$$

Here, $\hat{\mu }\left(t\right)$, ${\hat{\alpha }}_{e}\left(t\right)$, ${\hat{\psi }}_{1}\left(t\right)$ and their corresponding covariance structures were all estimated from real data. The background shared score $\xi_{i}$ was generated using the correlation structure of the LOCO null model covariance for the chromosome containing the target region and was rescaled to have the same marginal variance as ${\hat{\lambda }}_{1}$. The experiment-specific background process ${\tilde{D}}_{ie}\left(t\right)$ was generated from the experiment-specific covariance function estimated for experiment *e*.

The target region was fixed as the candidate window identified in the genome-wide analysis. To avoid choosing the simulated effect direction based on the observed phenotype association, the regional effect was injected only along the first local principal component of this window. Let $g_{i1}$ be the first local-PC score for rice line *i*, and define

(36) $$Q_{i}=\frac{g_{i1}-{\bar{g}}_{1}}{s_{g_{1}}}.$$

The nonzero effect magnitude was set to $\delta =\sqrt{0.05/0.95\;{\hat{\lambda }}_{1}}$. We considered three settings:

$$\begin{array}{cc} S0: & \;\delta_{Z}=0,\;\left(\delta_{D,1},\delta_{D,2},\delta_{D,3}\right)=\left(0,0,0\right),\\ S1: & \;\delta_{Z}=\delta ,\;\left(\delta_{D,1},\delta_{D,2},\delta_{D,3}\right)=\left(0,0,0\right),\\ S2: & \;\delta_{Z}=0,\;\left(\delta_{D,1},\delta_{D,2},\delta_{D,3}\right)=\left(0,0,\delta \right). \end{array}$$

Under S0, the target region had no effect. Under S1, the target-region effect entered the shared random function $Z_{i}^{*}\left(t\right)$ and therefore acted across all three experiments. Under S2, the shared random function contained no target-region effect, and the effect was introduced only into the E3-specific component $D_{i3}^{*}\left(t\right)$. Consistent with Section 2.3, this component was interpreted as an experiment-specific composite deviation rather than as a pure environmental or gene-by-environment effect.

For each setting, we performed 500 simulations and calculated the corresponding empirical rejection proportion. The nominal significance level was fixed at α=0.05. In every simulation, the shared functional representation was re-estimated. The prespecified target region was then tested using the genomic region association procedure, and the cross-experiment effect comparisons were repeated. The genome-wide scan itself was not repeated. All data processing, statistical analyses, and semisimulation studies were performed in R version 4.4.2 [34].

### 2.8. Sensitivity Analyses

We performed supplementary analyses to examine the sensitivity of the main regional association result to the phenotype representation and to the adjustment for population structure and genetic relatedness.

For the phenotype-representation sensitivity analysis, we averaged the three longitudinal curves for each rice line after removing the estimated experiment fixed effects and performed FPCA on the resulting mean curves. The corresponding principal component scores were then used in place of the shared functional principal component scores from Section 2.3 in the regional association analysis.

We also changed the number of global genotype PCs from the initial 5 to 3 or 10, reconstructed the chromosome-specific LOCO genome relation matrix using LD-pruned markers, and fitted a model without the LOCO polygenic random effect while retaining global principal component correction. Except for the settings under examination, the principal window definitions, local-PC representation, and multiple testing procedures remained unchanged.

As an alternative phenotype benchmark, we used the equal-weight average of the mean $\log_{10}$(PSA) values from E1, E2, and E3 repeats at relative day 20. The endpoint scan used the same 1673 primary windows, five global PCs, and chromosome-specific fully unique marker LOCO GRM as the main analysis, with chromosome-specific REML null models refitted for the endpoint response and Benjamini–Hochberg adjustment applied across all 1673 windows. Spearman correlation of window rankings and overlap between the top 10, 20, 50, and 100 windows were used to summarize genome-wide consistency with shared functional analyses.

## 3. Results

**Figure 1:**
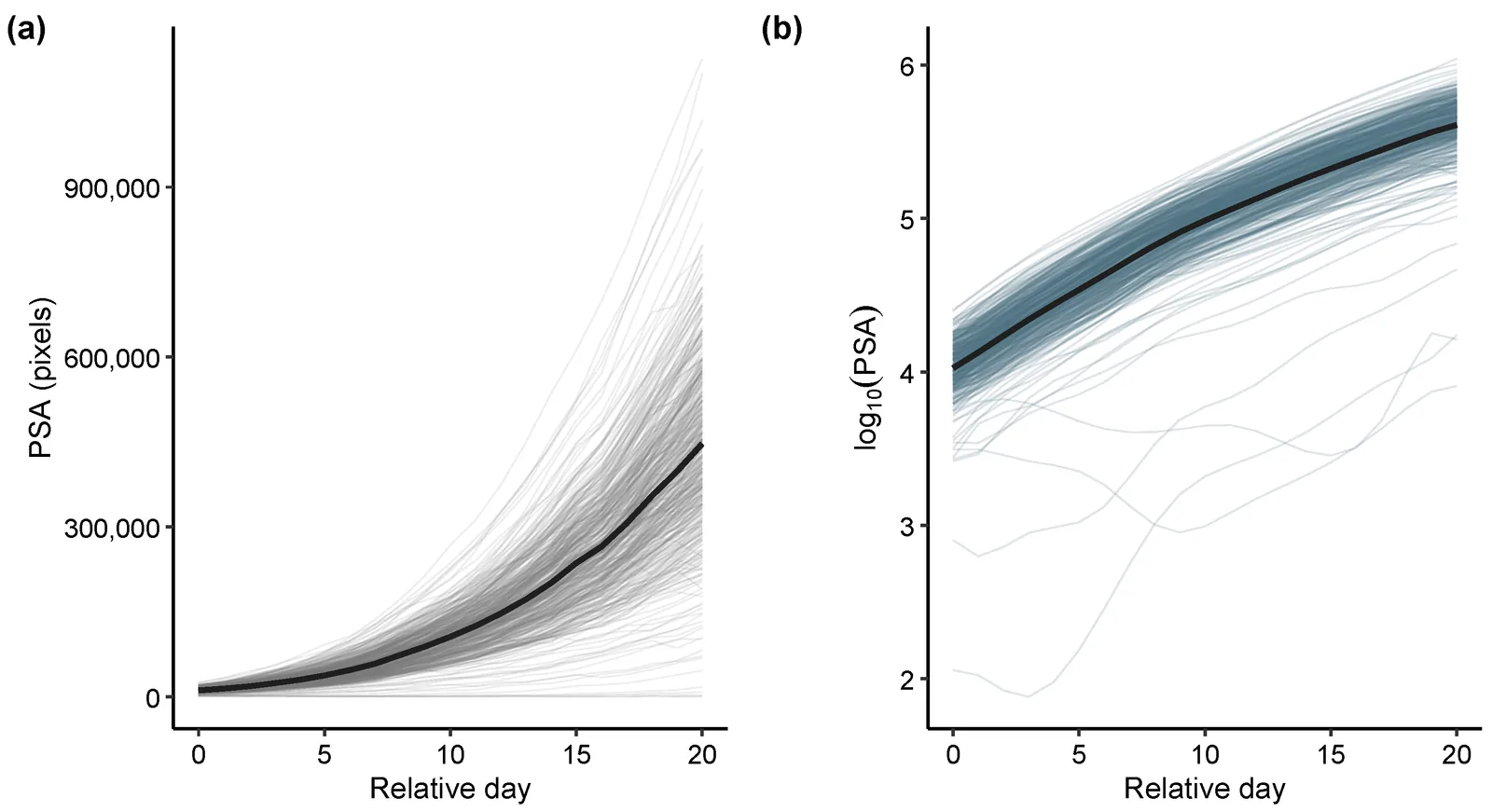
Longitudinal trajectories of projected shoot area in experiment E1. (**a**) Raw PSA trajectories. Thin gray curves represent individual plants and the thick dark curve is the pointwise mean. (**b**) Smoothed $\log_{10}\left(\mathrm{PSA}\right)$ curves. Thin curves represent rice lines and the thick dark curve is the pointwise mean.

**Figure 2:**
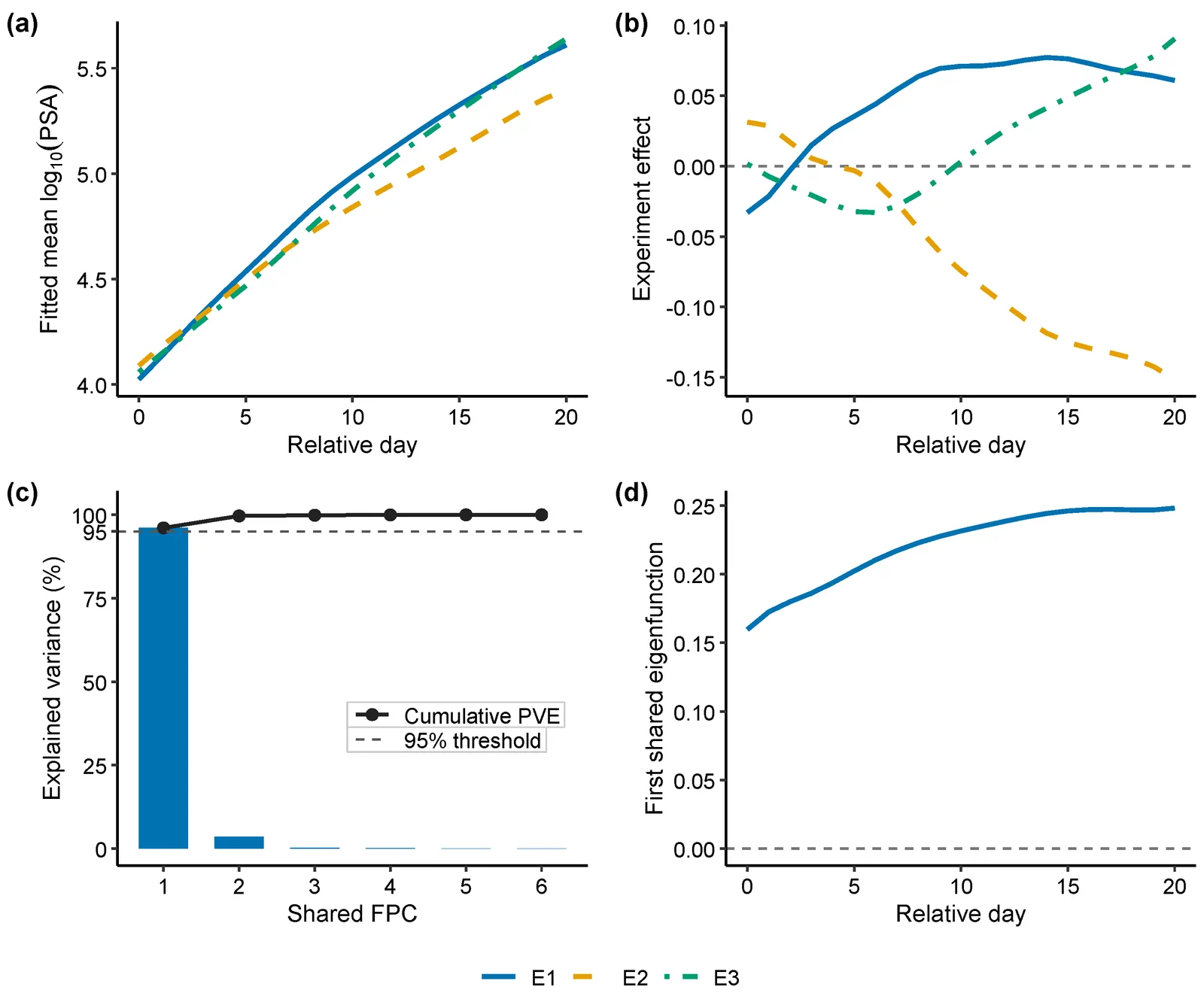
Shared functional representation of longitudinal phenotypes across experiments. (**a**) Fitted experiment-specific mean functions. (**b**) Experiment-effect functions. (**c**) Explained variance of the shared functional principal components and cumulative proportion of variance explained (PVE). (**d**) Estimated eigenfunction of the first shared functional principal component, ${\hat{\psi }}_{1}\left(t\right)$.

After phenotypic quality control and genotypic matching, a total of 356 rice lines were included in the subsequent analyses. Figure 1 shows the longitudinal PSA trajectories in experiment E1. As shown in Figure 1a, the raw PSA increased markedly over relative day, and the absolute differences among plants also increased over time. After logarithmic transformation, within-experiment replicate aggregation, and smoothing, the longitudinal curves of each line retained clear between-line variation while showing smoother temporal trajectories (Figure 1b). We then decomposed the longitudinal phenotypes from the three experiments. It can be observed that the overall growth trends of E1, E2, and E3 are relatively similar; however, the experimental effects vary over time, with E2 exhibiting a relatively lower experimental offset in the middle and late interval (Figure 2a,b). We performed FPCA on the shared covariance across experiments. The results showed that the eigenvalue of the first shared functional principal component (FPC) was ${\hat{\lambda }}_{1}=0.274$, explaining 96.069% of the shared variance. Accordingly, only the first shared FPC was retained for the genomic regional association analysis. Its eigenfunction varied smoothly over the full relative-day interval and had larger absolute loadings at later relative days (Figure 2c,d). The distribution of the first shared FPC scores and the estimated shared covariance surface are shown in Figure S2.

To characterize the complete functional covariance eigenspectra, we calculated the Shannon entropy, normalized spectral entropy, and entropy-based effective rank for the shared and three experiment-specific components, as shown in Table 1. The shared component had a Shannon entropy of 0.181 nats, a normalized spectral entropy of 0.101, and an effective rank of 1.198. The normalized spectral entropies of the experimentally specific components E1, E2, and E3 were 0.133, 0.201, and 0.567, respectively, with corresponding effective ranks of 1.357, 1.590, and 3.478. Among the four estimated covariance components, the shared component had the most concentrated eigenspectrum, whereas the E3-specific component distributed variation across more functional directions. The E1 and E2 specific components further illustrate the complementarity between leading PVE and the complete eigenspectrum. Their first-order FPC PVEs were relatively similar, at 0.928 and 0.902, whereas their conditional tail effective ranks were 1.928 and 4.311, respectively. The middle third of the relative daily interval contained 11.6% of the integrated tail variance for E1 and 22.0% for E2 (Figure S8; Table S6). These findings suggest that similar concentrations of dominant modes can coexist with different dispersion and temporal allocation of variation across secondary functional directions. The complete positive eigenspectra are shown in Figure S7.

**Figure 3:**
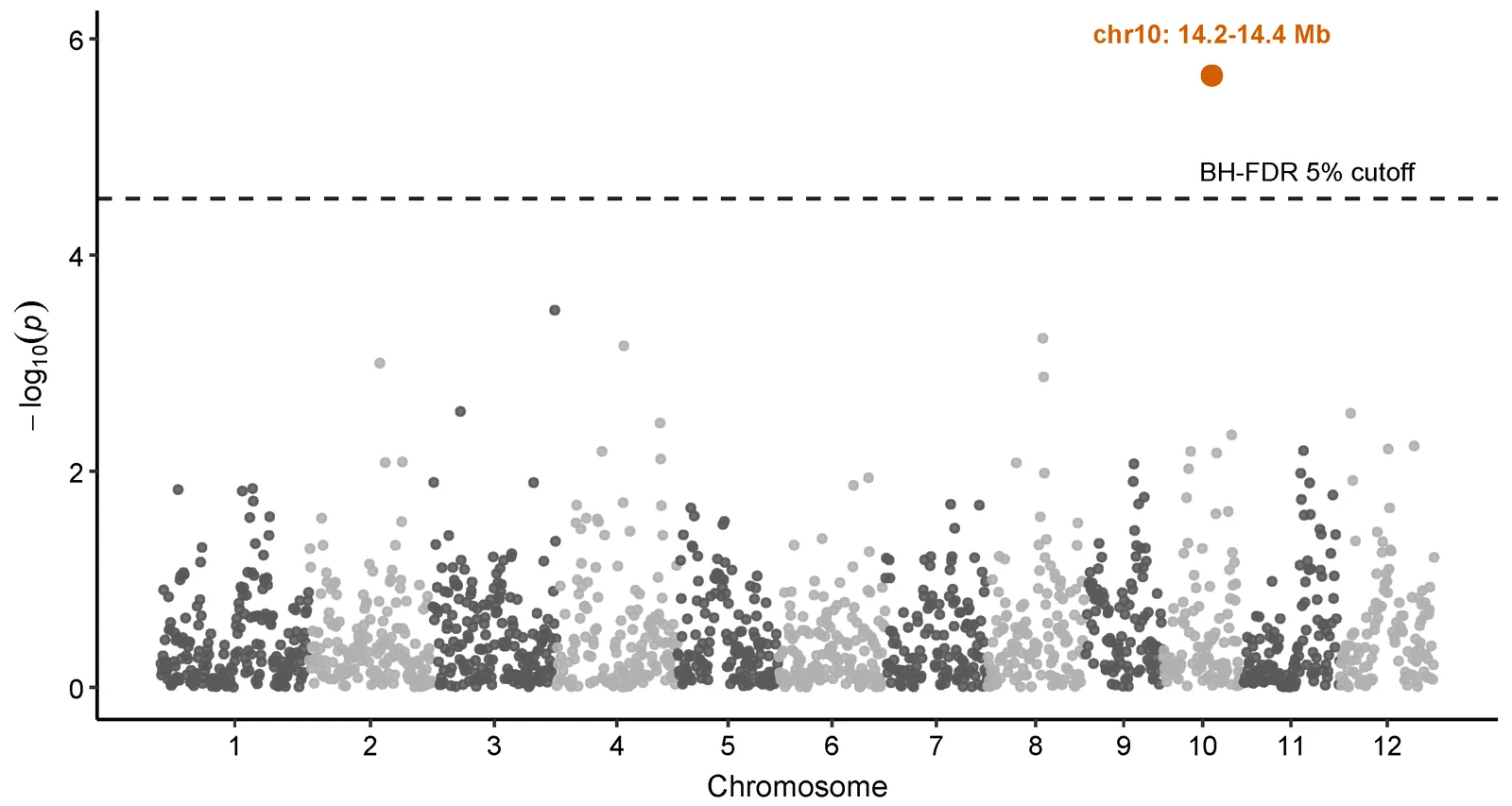
Primary genome-wide regional scan. Each point represents a 200 kb primary analysis window, and the vertical axis shows the corresponding $-\log_{10}\left(p\right)$ value from the regional Wald test. The dashed line marks the 5% Benjamini–Hochberg false discovery rate (BH-FDR) threshold, and the candidate window is highlighted.

**Figure 4:**
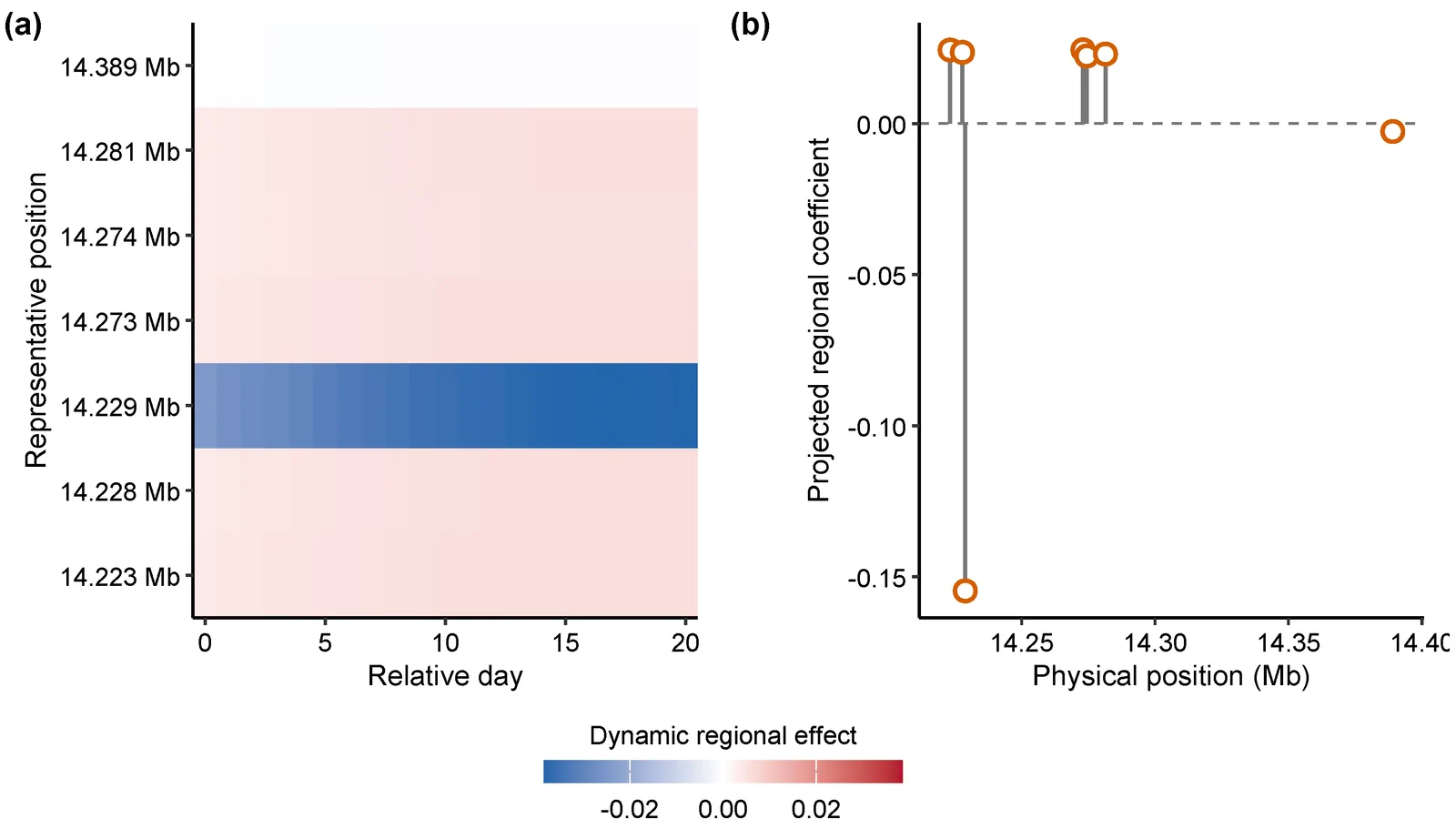
Dynamic regional effects of the chromosome 10 candidate region. (**a**) Dynamic regional effects at seven representative genomic position anchors. The diverging scale is centered at zero. (**b**) Projected regional coefficients at the seven representative positions.

Using the shared functional representation across experiments, we next performed regional Wald tests for 1673 non-overlapping 200 kb primary windows across the entire genome. The chromosome 10 (chr10) 14.2–14.4 Mb window was the only region that remained significant after Benjamini–Hochberg FDR correction, with Q=26.075, effective degrees of freedom =2, raw $p=2.177\times 10^{-6}$, and BH-adjusted q=0.004 (Figure 3). This window contained 8 quality controlled SNPs and 7 unique genotype vectors after removing identical genotype patterns. The local PCA ultimately retained 2 principal components, which collectively explained 90.810% of the genetic variation in that region, whereas the remaining windows did not reach statistical significance after FDR correction (Table S3). Building upon this, we projected the local-PC effects of this candidate region back to the seven representative genomic positions and combined them with the shared functional principal component to reconstruct the dynamic regional effects. Figure 4 shows that the projected regional coefficients differed in both direction and magnitude across positions. The projected regional coefficient at position 14.229 Mb had the largest absolute value (−0.155). Because only one shared FPC was retained, the reconstructed dynamic effects followed the same main temporal direction across positions, while their magnitudes and signs were determined by the corresponding projected regional coefficients. Supplementary Data S1 and S2 contain the complete window analysis results and the dynamic effect values for the chr10 region, respectively.

To place this statistical association in a biological context, we next examined the annotated genes within the chr10 candidate window and compared the region with previously reported rice QTL/GWAS signals. Using MSU RGAP version 6.1 to annotate the candidate window on chr10 spanning 14.2–14.4 Mb, we identified 36 overlapping gene models, including 31 protein-coding or predicted models and 5 transposon-associated models. None had locus-specific direct functional validation for rice shoot growth; 5 had rice association or expression evidence and 3 had only homology or pathway level evidence (Table S4). Compared with published rice QTL/GWAS results, no coordinate-verified growth-related signals were found that directly overlapped with the candidate window. The nearest published chr10 PSA QTL was 997,674 bp from the nearest boundary of the current window and was therefore classified as nearby rather than overlapping evidence (Table S5).

**Figure 5:**
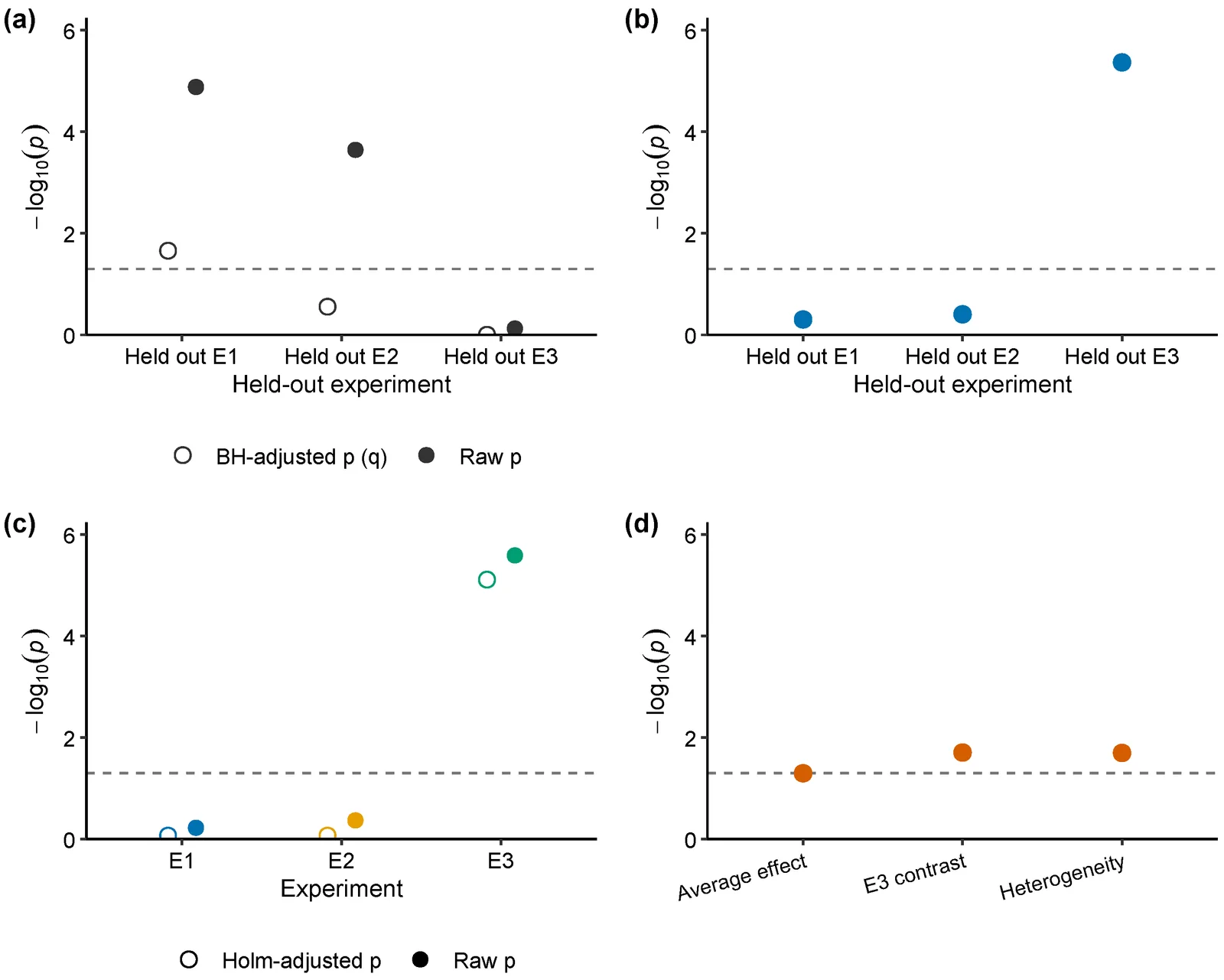
Cross-experiment association and heterogeneity assessments. (**a**) Regional association results from the leave-one-experiment-out (LOEO) analysis. (**b**) Targeted held-out assessment results in each LOEO experiment. (**c**) Experiment-specific regional association tests for E1, E2, and E3. (**d**) Average-effect, experiment-heterogeneity, and E3 contrast tests from the joint-experiment model. Dashed horizontal lines mark p=0.05.

We also conducted leave-one-experiment-out analysis as well as tests for experiment specific and combined effects to further examine the performance of the chromosome 10 candidate region across experiments. The results show that the association evidence was not fully consistent across experiments (Figure 5). The *p*-value for the region-specific analysis of E3 was $2.580\times 10^{-6}$, which remained statistically significant after Holm correction. In contrast, E1 and E2 did not show association evidence of the same magnitude. In the joint-experiment model, the average-effect test gave p=0.050, while both the experiment-heterogeneity test and the E3 contrast test gave p=0.020 (Figure 5d). These results indicate heterogeneity in the statistical evidence across the three experiments, with relatively stronger evidence in E3.

Beyond cross-experiment consistency, we evaluated the statistical calibration and robustness of the primary association from several complementary perspectives.Genome-wide calibration diagnostics showed no clear broad systematic inflation across the 1673 primary-window *p*-values. Across all 1673 windows, the median *p*-value was 0.474, and 5.56% of the *p*-values were at or below 0.05. The overall and stratified QQ diagrams by effective degrees of freedom are largely consistent with this pattern, although there are some degree-of-freedom specific tail biases (Figure S5).In the candidate specific conditional parametric bootstrap, none of the 20,000 null replicates reached the observed statistic $Q_{\mathrm{obs}}=26.075$, giving a plus-one empirical *p*-value of $5.000\times 10^{-5}$ (Figure S3a). Across the 356 leave-one-rice-line-out omissions, the candidate-window *p*-values ranged from $8.552\times 10^{-7}$ to $2.545\times 10^{-5}$, with no reversal in the direction of the projected regional effect (Figure S3b). These indicate that the association was not driven by any single rice line. We also conducted sensitivity analyses and found that changing the number of global PCs or using LD-pruned SNPs to construct the LOCO relationship matrix did not change the high genome-wide ranking of chr10. In contrast, the genome-wide regional association results changed more clearly when a simple phenotype representation based on averaging across experiments was used or when the genetic-relatedness random effect was omitted (Figure S4). As a separate phenotype benchmark, we compared the shared-functional analysis with a single-endpoint analysis based on relative day 20. No significant BH-FDR window was generated relative to the day 20 endpoint benchmark (Figure S6). For the chr10 candidate, the endpoint analysis gave p=0.0706, q=0.760, and genome-wide rank 154. The Spearman correlation between the shared functional window ranking and the endpoint window ranking was 0.495, and two of the top 10 windows overlapped in both analyses (Table S7). This indicates that the chr10 signal was attenuated in the endpoint representation but was more prominent under the shared longitudinal representation in this dataset.

**Figure 6:**
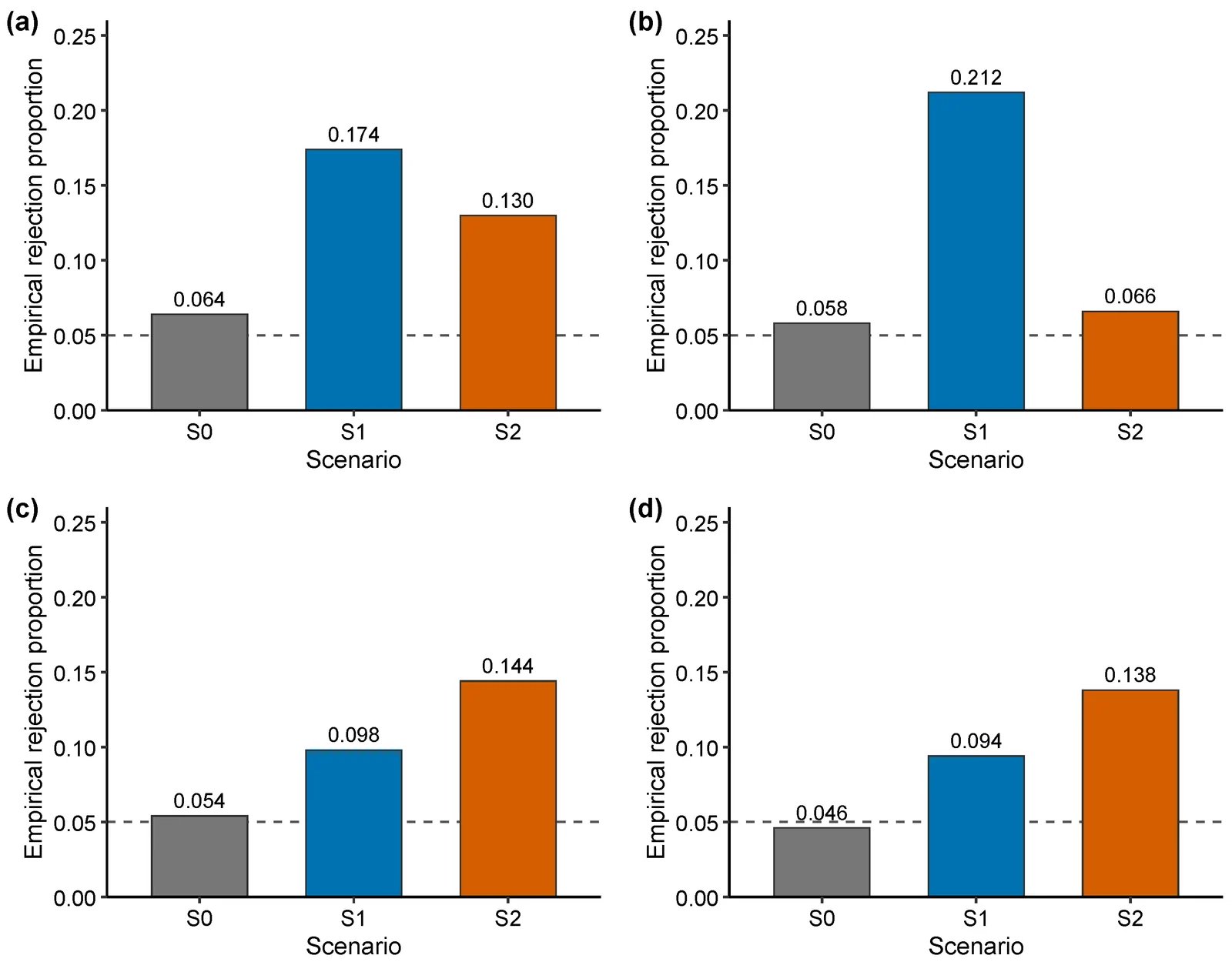
Empirical rejection rates of the tests in the semisimulation study. (**a**) Primary regional test. (**b**) Average-effect test. (**c**) Experiment-heterogeneity test. (**d**) E3 contrast test. Dashed lines mark the nominal 5% level.

Finally, we conducted a semisimulation study based on the observed genotype structure and estimated functional covariance to evaluate the empirical performance of the tests under different regional effect settings. As shown in Figure 6, in scenario S0 without a target-region effect, the empirical rejection rates for the main region test, average-effect test, experimental heterogeneity test, and E3 comparison test are 0.064, 0.058, 0.054, and 0.046, respectively, all close to the nominal significance level of 5%. Under the shared-effect setting S1, the rejection rates of the primary regional and average-effect tests increased to 0.174 and 0.212. Under the E3 only setting S2, the heterogeneity and E3-contrast tests had rejection rates of 0.144 and 0.138, respectively. Overall, within the three prespecified semisimulation settings, the four tests showed empirical sizes close to the nominal level under S0 and changes in rejection rates in the expected directions under the corresponding shared or E3-specific effect structures.

## 4. Discussion

Longitudinal high-throughput phenotyping can continuously capture plant growth and development. However, in multiple independent experiments, plant growth varies not only among different lines but is also affected by experimental background and other intra-experimental random variations. Directly performing association analysis at a single time point, or simply averaging different experiments, may overlook the continuous structure of longitudinal phenotypes and their cross-experimental information. Building upon existing functional regional association analysis approaches, we decomposed the longitudinal $\log_{10}\left(\mathrm{PSA}\right)$ curves from three independent experiments into the overall mean, experiment fixed effects, the shared random component across experiments, and experiment-specific deviations. We used the cross-covariance between experiments to extract a shared functional representation. The first shared FPC explained 96.069% of the shared variation, indicating that the shared differences in longitudinal growth among rice lines across experiments were largely concentrated along a single low-dimensional functional direction. The information-theoretic results based on the complete positive eigenspectrum further supported this low-dimensional structure. The shared component had a normalized spectral entropy of 0.101 and an entropy-based effective rank of 1.198, consistent with the strong concentration indicated by the high PVE of the first shared FPC. Spectral entropy and effective rank are not intended to replace leading PVE; instead, they summarize how variation is distributed across the complete normalized eigenspectrum. The E1 and E2 specific components provide a direct example of this complementarity. Their leading PVEs were both high and relatively similar, while the residual spectrum of E2 was more dispersed across secondary functional directions and showed a different temporal allocation of tail variation. This means that leading PVE mainly describes concentration in the dominant direction, and entropy-based quantities provide additional information about the distribution of the remaining variation. Similar information-theoretic metrics based on feature spectra have also been used to study the covariance structure of biological phenotypes [16]. Subsequently, we characterized regional genetic variation using a 200 kb local window and adjusted for global population structure and LOCO genetic relatedness, thereby linking longitudinal phenotypic information shared across experiments with regional genetic variation. Li et al. [18] developed a functional regional association framework for longitudinal quantitative traits using chromosome-level regions. In contrast, our analysis further considered shared functional variation across repeated experiments and examined regional associations at a finer 200 kb scale. In this way, the analysis combines shared longitudinal representation with localized regional association while retaining the joint nature of region-based testing.

Among the 1673 primary analysis windows across the genome, we identified one candidate region that remained significant after FDR correction, located at 14.2–14.4 Mb on chromosome 10. The joint genetic variation in this region showed a significant statistical association with the main shared growth pattern extracted from the three experiments. We further projected the local-PC regional effects back to representative genomic locations, finding that the projection coefficients at different locations exhibited different directions and magnitudes, forming a dynamic regional effect that changes over time along the principal components of the shared function. Since these quantities are projections of regional local-PC effects, they should be interpreted as region-level representations rather than as independent SNP effects. Biological annotation of the candidate window also supports a cautious interpretation. None of the 36 MSU6.1 gene models had locus-specific direct functional validation for rice shoot growth. A few gene models have evidence at the association, expression, or pathway levels, potentially serving as targets for future research, but current evidence does not support designation of a causal gene (Table S4). Comparison with published rice QTL/GWAS findings likewise identified no coordinate-confirmed growth-related signal directly overlapping the current window. The nearest published chr10 PSA QTL was approximately 0.998 Mb away and should therefore be regarded as nearby evidence rather than as overlap or replication (Table S5). Accordingly, the 14.2–14.4 Mb region on chromosome 10 requires further fine mapping and functional follow-up.

We also examined the association of the chromosome 10 candidate region across the three experiments. Results showed that E3 provided relatively stronger statistical support for this region, while the joint experimental model also indicated that the regional effects were not entirely consistent across the three experiments. Meanwhile, the normalized spectral entropy of the experimentally specific components of E3 was 0.567, and the effective rank was 3.478, both numerically higher than those of the experimentally specific components of E1 and E2. This indicates that the phenotypic variation not captured by the shared component in E3 was distributed across more functional directions and had a more dispersed eigenspectrum. The shared functional representation describes the main growth variation among rice lines that can be repeatedly observed across experiments; however, this shared phenotype direction does not require a candidate region to have exactly the same effect in every experiment. After replicate aggregation, $D_{ie}\left(t\right)$ represents an experiment-specific composite deviation that may contain line by experiment variation and remaining aggregated random variation. As the current data do not provide specific environmental variables, we cannot directly attribute these between-experiment differences to specific gene–environment interactions. The LOEO analysis and inter-experimental heterogeneity testing provided complementary information on cross-experiment robustness and heterogeneity. The semisimulation results also show that the prespecified tests responded differently under shared effect and E3-specific effect settings, indicating that the primary regional test and the cross-experiment-heterogeneity assessments provide complementary information.

The finite-sample and sensitivity analyses provided additional information on the stability of the primary finding. Genome-wide QQ diagnostics showed no clear broad systematic inflation. The candidate specific bootstrap and leave-one-line-out analyses also did not suggest that the association was driven by a single rice line. Even after altering some population structures and LOCO construction settings, the chromosome 10 candidate region maintained a high genome-wide ranking. Omitting the LOCO genetic-relatedness random effect led to significant changes in genome-wide association patterns, highlighting the importance of relatedness adjustment within this rice panel. Phenotype representation also affected the association results. Its association signal was weaker when the three experiments were summarized by simple averaging, and was further attenuated in the relative day 20 endpoint benchmark, in which no window remained significant after FDR correction. The shared longitudinal representation and a single endpoint summarize different aspects of the phenotype, which may lead to different genome-wide association patterns. In the present dataset, the chromosome 10 association was more prominent under the shared longitudinal representation than under the endpoint benchmark.

This study still has several limitations. First, both the phenotype and genotype data were obtained from existing public resources. The present analysis started from the derived PSA values provided by the source study and did not reprocess the raw RGB images. It therefore depends on the available metadata and upstream processing of those resources. Second, all three experiments were conducted on the same RDP1 panel. This design is useful for studying shared variation across repeated experiments, but cannot provide independent population validation. While the 200 kb windows and local PCA reduce local collinearity and enable joint regional testing, they also limit the direct interpretation of individual variants. Furthermore, the observed experimental differences could not be directly explained due to the lack of explicit environmental variables. Future work should examine the chromosome 10 candidate region in independent rice populations and new experiments, together with denser genotype or sequencing data, recorded environmental variables, and functional studies to refine the interval and evaluate its biological relevance.

## 5. Conclusions

In this study, we developed an integrated genomic regional association framework for longitudinal rice phenotypes measured across multiple experiments and used a shared functional representation to capture the major phenotypic variation common to the experiments. Among 356 RDP1 rice lines observed in three experiments, the first shared FPC explained 96.069% of the shared variation, while the normalized spectral entropy of 0.101 and entropy-based effective rank of 1.198 further indicated a highly concentrated shared covariance structure. Combined with local genotype dimensionality reduction, global population structure adjustment, and LOCO genetic relatedness, we identified a candidate region at 14.2–14.4 Mb on chromosome 10 and further characterized the dynamic regional effects of this region and its performance in different experiments. Our study results illustrate how shared longitudinal phenotypic variation, its covariance eigenspectrum, and local genomic variation can be analyzed within an integrated regional association framework. The specific genetic basis and biological relevance of the chromosome 10 region require further investigation in independent populations and functional studies.

## Figures and Tables

**Table 1:** Information-theoretic characterization of the shared and experiment-specific functional covariance components.

Component	$R_{A}$	$p_{A1}$	$H_{A}$ (nats)	$H_{A}^{\mathrm{norm}}$	$r_{\mathrm{eff},A}$
Shared	6	0.961	0.181	0.101	1.198
E1-specific	10	0.928	0.305	0.133	1.357
E2-specific	10	0.902	0.464	0.201	1.590
E3-specific	9	0.656	1.247	0.567	3.478

Note: $R_{A}$ denotes the number of positive eigenvalues for component *A*; $p_{A1}$ is the proportion of covariance variation explained by its leading eigenvalue; $H_{A}$ is the Shannon entropy in nats; $H_{A}^{\mathrm{norm}}$ is the normalized spectral entropy; and $r_{\mathrm{eff},A}$ is the entropy-based effective rank.

## Data Availability

The original projected shoot area (PSA) data are openly available at Zenodo (https://doi.org/10.5281/zenodo.1313684), and the rice genotype data are available from the Rice Diversity Project (http://ricediversity.org). The derived data supporting the analyses reported in this study are provided in the Supplementary Materials (Data S1–S4).

## Supplementary Materials

The following supporting information can be downloaded at https://www.mdpi.com/article/10.3390/e28101091/s1. Figure S1: Window and population-structure diagnostics; Figure S2: Shared functional diagnostics; Figure S3: Finite-sample diagnostics; Figure S4: Sensitivity analyses; Figure S5: Genome-wide *p*-value calibration diagnostics; Figure S6: Endpoint benchmark; Figure S7: Complete positive functional eigenspectra; Figure S8: PVE–entropy complementarity; Table S1: Analysis design and cohort summary; Table S2: Shared functional principal components; Table S3: Top 20 primary-window association results; Table S4: Gene models in the chr10 14.2–14.4 Mb candidate interval; Table S5: Comparison with published QTL and GWAS results; Table S6: Spectral-tail diagnostics; Table S7: Shared-functional versus endpoint benchmark; Data S1: Primary 1673-window regional scan; Data S2: chr10 dynamic regional effects; Data S3: Endpoint 1673-window regional scan; Data S4: Complete positive eigenspectra and entropy decomposition.

## Abbreviations

The following abbreviations are used in this manuscript:

BH	Benjamini–Hochberg
chr	Chromosome
FDR	False discovery rate
FPC	Functional principal component
FPCA	Functional principal component analysis
GRM	Genomic relationship matrix
GWAS	Genome-wide association study
LD	Linkage disequilibrium
LOCO	Leave-one-chromosome-out
LOEO	Leave-one-experiment-out
MAF	Minor allele frequency
PC	Principal component
PCA	Principal component analysis
PSA	Projected shoot area
PVE	Proportion of variance explained
QQ	Quantile–quantile
QTL	Quantitative trait locus
RDP1	Rice Diversity Panel 1
REML	Restricted maximum likelihood
RGB	Red–green–blue
SNP	Single-nucleotide polymorphism
